# Sleep Paralysis and Lucid Dreaming—Between Waking and Dreaming: A Review about Two Extraordinary States

**DOI:** 10.3390/jcm12103437

**Published:** 2023-05-12

**Authors:** Severin Ableidinger, Brigitte Holzinger

**Affiliations:** 1 Institute for Consciousness and Dream Research, 1180 Vienna, Austria; 2 Sleep Coaching, Medical University Vienna, 1090 Vienna, Austria

**Keywords:** sleep paralysis, lucid dreams, REM sleep

## Abstract

Background: Sleep paralysis and lucid dreams are two states of consciousness that are connected to REM sleep but are defined by higher awareness in contrast to regular REM sleep. Despite these similarities, the two states differ widely in their emotional tone and their perceived controllability. This review aims to summarize the current research containing sleep paralysis and lucid dreams. However, given the sparsity of the research, one single topic cannot be chosen. Methods: Articles containing both lucid dreams as well as sleep paralysis were searched for in the following databanks: MEDLINE, Scopus, Web of Science, PsycInfo, PsycArticles, and PSYNDEX. Further, citations of the found papers were examined. Results: 10 studies were included in the review. Most of the studies were surveys, but there was also a case study, a randomized trial, and an observational EEG study. The numbers of participants ranged from a single participant in the case study to 1928 participants in a survey. The main findings were that correlations between sleep paralysis and lucid dreaming were positive and significant in most of the studies. Conclusions: There is a connection between lucid dreaming and sleep paralysis. However, research is still very limited and diverse in the methodologies used. Future research should build standardized methods for examining the two phenomena.

## 1. Introduction

Sleep paralysis (SP) is characterized as an inability to move while feeling fully awake and aware of your surroundings [1]. Though most muscles cannot be moved, reports often involve the ability to move the eyes. The eyes are usually closed during SP, but they can be opened [2]. SP happens, in most cases, upon waking up or when falling asleep. The experiencer is conscious and can afterwards describe the full event in detail. The inability to move is most often accompanied by fear and, often, frightful hallucinations. These can include figures, sounds, pressure, or the sense of a presence. Further, 75% of all SP episodes are estimated to contain these hallucinations [3]. The hallucinations are often thought to be pictures of dreams, which intrude into the waking perception. Recently, it was argued that SP does have some sort of “aura”. It was found that auditory, tactile, and visual perceptions may prelude SP [4].

SP is connected to rapid eye movement (REM) sleep. REM sleep is one of the stages of sleep, together with non-REM (NREM) sleep, which further consists of three separate sleep stages (N1, N2, and N3). REM sleep is characterized by fast, jerky eye movements and high dream recall when awakened from this sleep stage. Further, it is characterized by muscle atonia, which is thought to occur so that the experienced dreams are not acted out. As the brain is still sending signals to move the body according to the dream content, muscle atonia while in REM sleep is important, so as not to harm oneself or others while sleeping. This is theorized to be the reason that one cannot move during SP [1].

Cheyne and Girard [5] classified the occurring hallucinations into three categories: intruder, incubus, and vestibular-motor (V-M).

- Intruder hallucinations include the sense of a presence and sensory hallucinations, such as seeing figures, hearing footsteps, and the sensation that something is pulling on the bed sheets.
- Incubus hallucinations include difficulties with breathing, feeling pressure, most often on the chest, feelings of strangulation, choking, and feelings of impending death.
- V-M hallucinations involve feeling as if one is floating, falling, or flying, but also out-of-body experiences (OBEs), which can happen during SP, fall under this category. Autoscopy, which is seeing oneself from an external station point, and illusory motor movements, such as arm movements, sitting up, and moving around can also be experienced during SP.

OBEs are often treated as an entirely different phenomenon than SP and also have similarities to lucid dreaming (LD). Like in LD, in OBE, the experiencer can often move around freely, according to their own will. Further, V-M hallucinations are often accompanied by positive feelings, including bliss, which is often stated as a common feeling in LD and in OBEs. In this, the V-M hallucinations are different from intruder and incubus hallucinations, which are usually accompanied by fear. Intruder and incubus hallucinations can be isolated but usually co-occur. 

Different studies found different percentages on how much of the SP is actually connected to fear. The percentages range from 80 to 90% [1]. In the same range, different studies found similar percentages on how many episodes of SP were pleasant and included positive feelings, such as bliss. About 16% to 17% of participants seem to experience pleasant SP [6,7].

Occurrences of SP are well known throughout history and in different cultures. However, they are not uniformly called SP, but vary through different cultures, who found different names for the often-mythical creatures they made responsible for the episode. These creatures are most likely seen as accompanying hallucinated figures during the episodes. For example, in Italy, the Pandafeche shows up during episodes where one cannot move while lying in bed and feeling crushed; in Newfoundland, it is the “Old Hag”; the kanashibari in Japan; the Jinn in Egypt; in Nigerian culture, a female demon is blamed; and Canadian Eskimos say that spells of shamans cause SP [8,9]. Even the origin of the name nightmare is thought to be rooted in the word mare, which is some sort of goblin, which afflicts one in their sleep with the feeling of suffocation [3]. Further, alien abductions and UFO encounters can occur in SP, and it has been argued that at least a proportion of alien and UFO encounters can be explained by SP [10,11].

The physiology of SP resembles the physiological correlates of REM sleep. However, heart rate is most often elevated during SP [12], which is probably due to fear, as this can also be seen in nightmares [13]. Similarly, SP is also associated with nightmares [14]. However, it has also been argued that SP is a “mixed” state between REM sleep and wakefulness [2]. During SP episodes, EEG activity has been found with abundant alpha activity, which is normally associated with waking, mixed with more typical REM EEG activity [15,16].

### Lucid Dreams

LDs are dream states in which the dreamer is aware of being in a dream, and with this realization, they can choose to alter the content of the dream. However, it has been stated that only a subset of LDs involve control. Often in dreams, the dreamer wakes up upon realization that one is dreaming. In one study, only 37% of those who said to be lucid dreamers reported that they could manipulate their dreams [17]. It is estimated that about 55% of people have experienced one LD or more in their lifetime, and about 23% experience LDs about once a month or more often [18].

The term “lucid dreaming” itself was coined by Frederik Willems van Eeden [19]; however, the phenomenon was described a long time before that, and LD was used by Tibetan Buddhists, as well as in Sufism and Indian yoga [20]. 

LDs can also be used in therapeutic settings [21]. They are able to reduce suffering through nightmares, anxiety, and depression [22,23,24]. Further, LDs have found some use in scientific research. It has been found that using lucid dreamers can give prior agreed-on signals, which can be detected by researchers to detect LDs. For this, moving the eyes left, right, left, and right has been used, which can then be detected by a polygraph [25]. However, recently, a new technique has been proposed, which uses chin movements to indicate LDs and can be detected via EEG. This would allow for easier detection of LD, as this uses less equipment and might be easier on the participants [26]. It has also been found that signals can be sent into dreams, which can induce LDs [25]. Although LDs are praised as a way to combat nightmares, and as being full of bliss and positive emotions, Aviram and Soffer-Dudek [27] have shown that deliberate LD induction can lead to an increase in dissociation and schizotypy symptoms, and it has been argued that, in fact, LDs might not be recommendable for everybody [21]. 

LDs most often occur during REM sleep, although there have been reports of LDs in NREM sleep [28]. It has been proposed that LDs are a “hybrid” state, combining both elements of dreaming and waking [29]. There are various findings about how LDs differ from regular dreaming in neuronal activation [30]. Still, it has recently been argued that LDs are in fact happening in REM sleep and not in a mixed state [31].

Just like SP, LDs have also been argued to be responsible for at least some alien and UFO encounters and to be connected to the supernatural [32,33]. In monotheistic religions, dreams were often seen as being sent from God. One Christian philosopher viewed LDs as a kind of preview of the afterlife. Therefore, in this way, LDs also differ from SP, as they are sent from God, while SP is mostly the work of demons and spirits who want to cause harm [34]. 

Therefore, both LDs and SP are connected to REM sleep. Further, both are characterized by heightened awareness compared to regular dreaming. Further, both LDs and SP have been described as hybrid states between REM sleep and wakefulness, which are characterized by high cortical activity and mixed neuromodulation. In LD practitioners, it is common to use certain induction techniques to induce an LD. There have been reports that these techniques may also lead to SP [35]. Previous studies also showed that both LDs and SP are connected to creativity [36,37,38,39]. Further, both phenomena are connected to supernatural and extraordinary experiences. However, while LDs are mostly active, in SP, the experiencer can most often not move and cannot take an active role. Further, SP is often connected to fear, while LDs are usually filled with emotions such as bliss.

This review aims to summarize the current research containing both SP and LDs and to further assess the connections, similarities, and dissimilarities of these two phenomena. Therefore, we conducted a search for papers describing both LDs and SP. As seen below, there is limited literature dealing with the two phenomena, so we could not pick one specific topic concerning both SP and LDs but decided to provide an overview of the current literature. This also aims to show the limitations and shortcomings of the current research.

## 2. Methods

We searched the databanks MEDLINE, Scopus, Web of Science, PsycInfo, PsycArticles, and PSYNDEX. Search terms were “sleep paralysis” and “lucid” or “lucid dream*” or “lucid dreaming”. In Scopus, we searched in “Article title, Abstract, Keywords”. In MEDLINE, PsycInfo, PsycArticles, and PSYNDEX, we did not specify search fields, so Title, Abstract, Keyword, and other databank-specific fields were searched. In Web of Science, we searched “All Fields”, which involved Title, Abstract, Keywords, Categories, and others. No further search strategies were implemented in searching the databanks. While the search on Scopus was conducted on 11 January 2023, the other databases were searched on 16 January 2023. Further, citations of the found papers were examined for further papers, which were not found in the databanks. All studies were included that empirically researched both SP and LDs and reported measures involving both of them, such as correlation, or compared the measures between them. Due to the sparsity of research on those two phenomena, there were no further inclusion criteria. One reviewer searched the databases and decided for each study whether or not they fit the inclusion criteria. This was conducted by checking the reported results in each of the studies for the appropriate measures. All measures, including both SP and LDs, were extracted, which mainly involved correlations. Further, all measures, on which SP and LD were compared, such as factors that correlated with them, were extracted. Sample characteristics, such as size and gender distribution, were also examined as well as the sampling strategy, if reported. Finally, as far as they were reported, methodologies, such as measurement of SP frequency, were extracted. No automation tools were used for either study selection or data collection. Everything was extracted from the written reports, and no further data were obtained. As the studies had different methods and often used different measures, no synthesis of the results was undertaken. As far as possible, PRISMA guidelines were followed; however, as no synthesis could be performed and the studies differed in their measures and methods, many of the aspects were not relevant to this review [40].

## 3. Results

**Figure 1:**
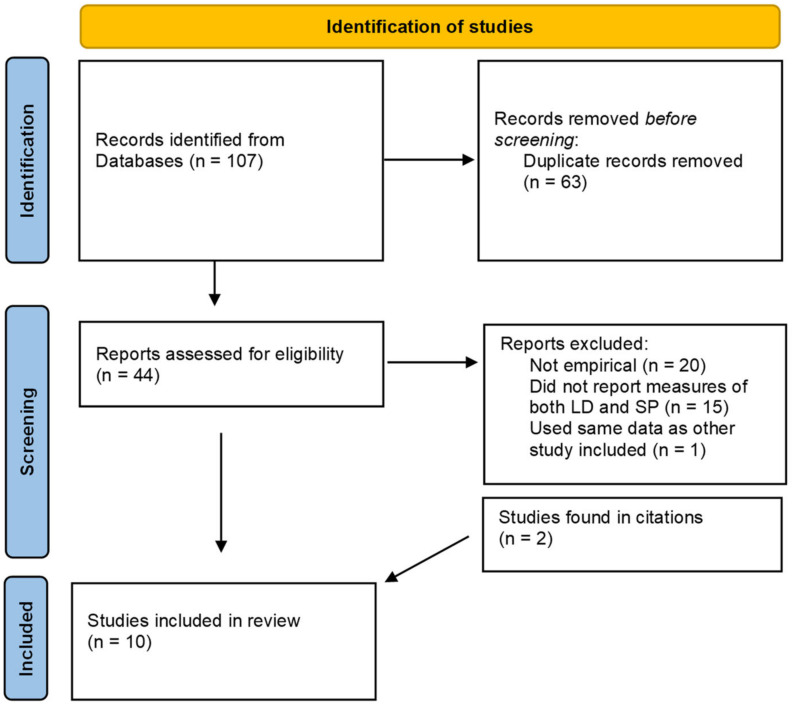
Flowchart of the identification of studies for the review [40]. LD: lucid dreaming; SP: sleep paralysis.

A total of 107 documents were found. After removing duplicates, a total of 44 documents remained. After excluding those documents that did not have any empirical measures, such as reviews and book chapters (*n* = 20), and those studies that did not report measures of both LDs and SP (*n* = 15), nine Studies were found, reporting correlations or other measures concerning both SP and LDs. One further study was excluded, as it used the same data as another study included in the set. Through citations of some of the found papers, we further found one patent application and another study whose results were only presented in a conference but not published otherwise. Therefore, in total, 10 studies were included (see Figure 1), with the results summarized below. All of the papers were written in English. 

Publication dates, including the patent and the year of the conference, ranged from 2002 to 2022. Seven of those studies were surveys, six of which were online surveys, and one was a life survey on the streets of Moscow. One of those further included a case study and individual reports. Further, there was one case–control study using interviews, one study using EEG measures, and a randomized, double-blind, placebo-controlled crossover trial. Therefore, of these 10 papers, only 1 included an experiment, while all the other papers purely included observational study designs.

Given the different methodologies, the numbers of samples vary largely. While the case study only had one subject, the author, the EEG study had five participants, and the randomized trial had ten volunteers. In the case–control study, 53 narcolepsy patients were compared to 53 healthy controls. The surveys ranged from 92 participants to 1928.

### 3.1. Short Description of the Studies

A study by Biehl [41] aimed to examine the relationship between food and substance intake and LDs. For this, a sample of 436 participants answered an online questionnaire. Further, 45.7% of the sample was female. Participants were recruited via social media groups and internet forums concerning the topic of LDs and the website klartraum.de. They were asked how often they experience LDs and SP on an eight-point rating scale ranging from “never” to “several times per night/day”. This was also asked for other sleep- and dream-related phenomena, such as dream recall frequency, hypnagogia recall frequency, nightmare frequency, pleasant dream frequency, and surreal dream frequency. The participants were further asked about food and substance intake for cereal products, dairy products, meat, fish, fruit, other foods containing sugar, protein supplements, vitamin supplements, chili, caffeine/theine, alcohol, nicotine, and antidepressants. Finally, regarding personality, the participants completed the NEO-FFI. It was found that LD and SP frequencies correlated with r = 0.276, *p* < 0.001. Further, both LDs and SP were correlated with dream recall frequency, hypnagogia recall frequency, nightmare frequency, and pleasant dream frequency, and both correlated with openness to experience and extraversion. There were no foods or substances that correlated with both LDs and SP.

In a paper by Conesa [42], regarding ten-year-long observations, the author wrote about his own dreamscape in the form of a longitudinal case study. Further, the results from an online questionnaire posted on a website, a survey, as well as various narratives, which were contributed from self-selected subjects, were presented. For the survey, psychology students were recruited, who did not know the purpose of the study. For the questionnaire and for the narratives, no data, which would correspond to the objective of this review, were published, so these are not further discussed. The questions for the survey were not published. In the ten-year-long case study, the author documented all his dreams and calculated how often certain phenomena corelated. This involved LDs and SP, which correlated with r = 0.31. The author states that he used a method with which he can use SP to move into an LD. Further, LDs correlated very highly with flying dreams with r = 0.81, while SP negatively correlated with ISP r = −0.29. In an online survey, 16.3% reported experiencing both LDs and SP, which were less than the group who only experienced LDs (50%), but more than the group reporting only SP (7.6%). 

A study by Denis and Poerio [43] aimed to find commonalities between LDs and SP. For this, they recruited 1928 participants in an online survey, 53% of which were female. The age of the participants ranged from 18 to 92 years, with a mean of 24.17, SD = 13.62. They were recruited through advertisements in university mailing lists, and on LD and SP websites and forums. Measurements included the Waterloo Unusual Sleep Experiences Questionnaire-Vlla, which measures SP with two questions, including frequency and intensity, on a 7-point rating scale, ranging from “never” to “several times a week” and “vague and suggestive” to “a very clear and distinct impression”. The two questions were then averaged. Frequency and intensity, with frequency ranging from “never” to “always” on a four-point scale, were also asked about, in terms of the three types of hallucinations (intrudes, incubus, and V-M hallucinations), with varying number of items for each (5, 4, and 8, respectively). These subscales were also averaged. LDs were asked about in a similar way to SP, with two seven-point rating scales for both frequency and intensity. Other measurements involved sleep quality, daydreaming frequency, positive constructive daydreaming, dissociative experiences, mindfulness, imagery, depression, anxiety, life stress, conspiracy beliefs, and paranormal beliefs. It was found that SP and LD frequency correlated significantly with r = 0.15, *p* < 0.001. LDs further correlated with intruder hallucination frequency (r = 0.08, *p* = 0.01; and intensity: r = 0.10, *p* = 0.01) and V-M hallucinations (frequency: r = 0.25, *p* < 0.001, intensity: r = 0.28, *p* < 0.001) but did not correlate with incubus hallucinations (frequency: r = −0.004, *p* = 0.89, intensity: r = 0.03, *p* = 0.41). SP and LD frequency were further correlated with daydreaming, positive constructive daydreaming, dissociative experiences, depression, anxiety, stress, and paranormal beliefs. However, depression, anxiety, and stress were positively correlated with SP but negatively correlated with LDs.

In the case–control study, patients with narcolepsy were compared to healthy individuals [44]. Thus, 53 narcolepsy patients were matched with 53 healthy controls, who were sampled via family, friends, hospital employees, and students. Of the narcolepsy patients, 41.5% were female, and 43.3% were female in the control group. The data were gathered via in-person interviews, which included questions about nighttime sleep characteristics, such as SP. Further data, such as the Epworth sleepiness scale, mean daytime sleep latency, and amount of sleep onset in REM periods, were taken from medical files in the narcolepsy patients. Further sleep characteristics that were asked about were evaluation of monthly frequency of dreams, nightmares, prominent emotions in dreams, dreams of false awakenings, recurrent dreams, enchained dreams, and LDs. The utility of LDs was also evaluated. Results from an LD experiment performed in a sleep laboratory were also reported. From the patients, 12 underwent nighttime and daytime sleep monitoring, as did 5 healthy controls, who were frequent lucid dreamers and were recruited via word of mouth. The results from this did not include any data of interest, so they are not further discussed here. The case–control study found that participants with narcolepsy had LDs more often than controls, 77.4% vs. 49.1% had LDs at any point in their life and 58.5% vs. 17% regularly had LDs. Further, patients with narcolepsy also had SP more often, 58.5% vs. 15.1% in controls. In the sample of narcolepsy patients, it was found that in the lucid dreamers, 56.1% had SP, and in those who did not dream lucidly (66.7%) had SP; however, the number of subjects here was rather small (41 and 12), and the difference was not significant. 

Drinkwater, Denovan, and Dagnall [45] aimed to find out the associations between reality testing deficits, paranormal experience, and nightmares, LDs, and SP. For this, 455 participants, 76% of whom were female, answered an online survey. The mean age was 34.46 years, SD = 15.7, and age ranged from 18 to 77. The participants were recruited via emails to university staff and students as well as to local businesses, leisure, and sports classes. LDs were measured via an eight-point rating scale, ranging from “never” to “several times a week”. Further, participants rated the extent they were able to maintain conscious awareness, completely control their dream body, and design their dream surroundings (as a percentage). Recall of SP was measured via a four-point rating scale, ranging from “never” to “more than five times”. Nightmares were measured as well on an eight-point rating scale for frequency and a five-point scale for nightmare distress. Further, reality testing deficits were measured, as well as paranormal beliefs and experience. LD and SP frequency correlated significantly with r = 0.23, *p* < 0.01. The two measures were further connected to nightmare frequency, paranormal experience, delusional thinking, and reality testing.

Kliková, Sharpless, and Bušková [46] examined pleasant SP, meaning those occurrences of SP that are not filled with fear but instead with positive feelings and bliss. Thus, 172 participants volunteered for an online survey, consisting of 68% women, with a mean age of 23.7, SD = 5.5. These were recruited via social media networks, university groups, and online organizations, which were interested in SP. A questionnaire was created with seven items on episodes of SP and on LDs. The frequency of SP was assessed with an eight-point rating scale, ranging from “never” to “daily”, and the occurrence of LDs was assessed with a simple yes-or-no question, as well as the ability to induce LDs with a yes-or-no question. Pleasant episodes of SP were assessed with various questions, including a yes-or-no question and a question regarding frequency on a four-point rating scale, ranging from “never” to “always”. Further, trauma symptoms and life satisfaction were assessed, and personality was measured in the form of the BFI-44. Data on a connection between LDs and SP, in general, were not reported, but positive associations between pleasant episodes of SP and LDs were found via a chi-squared test with X2 (1, N = 172) = 8.414, *p* = 0.004, and φ = 0.22, indicating a small to medium effect size as well as an association between pleasant SP and the ability to induce LDs, with X2 (1, N = 134) = 9.327, *p* = 0.002, and φ = 0.26. In the study, they also found that, although 23% had experienced pleasant SP, fear was present in about 97% of the participants in SP regularly, and only about 3% had never experienced fear in SP; in 54.5%, SP always included fear.

The patent application publication [47] used a randomized, double-blind, placebo-controlled crossover trial to assess the effect of donepezil on LDs. This was carried out with 10 participants, of which 30% were female and the age ranged from 22 to 55. They collected dream content and other self-reported measures on three nights with washout periods in between. At bedtime, they took 0 (as a placebo), 5, or 10 mg of donepezil. An effect of donepezil on lucid dreams was found: 9 out of the 10 participants reported one or more LD on nights with donepezil, while only one participant reported a lucid dream on a control night (placebo). Donepezil was also associated with an elevated frequency of SP, but no exact data were provided here. It is not further explained if this means that 9 out of the 10 participants had a lucid dream on both nights with donepezil or on at least one of those two nights.

Mainieri et al. [48] used EEG measures to examine SP, LDs, false awakenings, and REM sleep. Five participants underwent examination in a sleep laboratory, 80% of whom were female. From this, five episodes of SP were captured. Two episodes were marked with the ocular left–right–left–right code, which is normally used to signal LDs. These two episodes were from the same participant. One episode, from a different participant, was marked by an external noise, and two were retrospectively reported. Further, two false awakenings were registered, and in four out of these five participants, normal REM sleep and wakefulness were also analyzed. The recordings were further compared to recordings of LDs in four different patients with narcolepsy. All episodes of SP happened during REM sleep, and almost all in sleep-onset REM. During SP, increased amounts of EEG alpha rhythms were found compared to lucid REM sleep. In lucid REM sleep, more enhanced muscle tone was found than during SP. 

Raduga, Kuyava, and Sevcenko [11] aimed at finding relations between various phenomena, involving LDs, SP, OBEs, and false awakenings. For this, data from 974 participants, 54% of whom were female, were gathered in a live survey on the streets of Moscow. The age ranged from 10 to 87, with a mean age of 29, SD = 15. In the survey, information was gathered about sleep duration in hours, dream recall frequency, LDs, false awakenings, SP, OBEs, and overall awareness about practices, such as LD and astral projection. For sleep duration, dream recall frequency, LDs, false awakenings, SP, and OBEs, they were asked how often they experience these on a four-point rating scale, ranging from “never” to “often”, with the option to not answer. For the analysis, they compared those who often experience a phenomenon to those that do not experience it. Differences were found between participants who often experience LDs and those who do not experience them in terms of whether they often experience SP. In participants who often experience LDs, 5% often experience SP and 70% experience no SP; in participants who do not experience LDs, only 1% experience SP often and 83% do not experience SP. A chi-square test prompted a significant difference X2 (25, N = 974) = 126.767, *p* < 0.001. Phi was not reported but can be calculated using the given test statistics and equals φ = 0.36, which indicates a medium effect size. Both SP and LDs were further connected to dream recall frequency, false awakenings, and OBEs. There was a significant connection between LDs and age and a trend between SP and age, but there was no significant connection. In both, older participants reported fewer episodes of either LDs or SP. SP was significantly connected to sleep time, with participants sleeping less than 6 h or more than 9 h reporting more SP. This trend was also seen in LDs, but there was no significant connection.

Solomonova, Nielsen, and Stenstrom [49] examined the relationship between LDs, SP, and nightmares via an online questionnaire. Thus, 245 participants, of whom 58% were female, with a mean age of 30.9, SD = 13.5, filled out the survey. In this, they gave information about frequency of LDs, SP, and nightmares on a seven-point rating scale and about SP and nightmare distress on a five-point rating scale. LDs were correlated with SP frequency r = 0.24, *p* < 0.001, and SP distress r = 0.21, *p* = 0.001, but not with nightmares or nightmare distress. Correlations of nightmares and SP were not reported. All the summaries can, in short, be seen in Table 1.

### 3.2. Correlation and Cooccurrence of SP and LDs

Correlations between SP and LDs were positive and significant in almost all the studies. 

Frequencies of SP and LD correlated at 0.15 [43], 0.24 [49], or 0.276 [41]. In Raduga, Kuyava, and Sevcenko [11], 5% of participants often had SP and LDs; in Conesa [42], 16.3% of participants reported both LDs and SP; and in Dodet et al. [48], 56.1% of regular lucid dreamers with narcolepsy also had SP. In both Raduga, Kuyava, and Sevcenko [11] and Conesa [42], there where more than those who only had SP, 1%, and in Raduga, Kuyava, and Sevcenko [11], and 7.6% in Conesa [42]. In Dodet et al. [48], these were less than those who only had SP—66.7%; however, the sample sizes for these two groups were rather small, 41 and 12, but this difference was not significant. Still, this would suggest a trend in the opposite direction to other papers. There could be a difference in narcolepsy, but this could also be a statistical artifact. Conesa [42] further reported that, in his personal recordings, SP and LDs correlated with 0.31, and he explained that he used SP as a “launch pad” to move into LDs.

Solomonova, Nielsen, and Stenstrom [49] showed that LD is not only related to SP frequency but also to SP distress. Denis and Poerio [43] showed, further, that the relationship between LDs and SP V-M hallucinations was higher than the relationship of LDs to any other SP hallucination. 

Given the differences in the studies and in methodologies, for example, no two of these studies used the same questions for LDs and SP, these different estimates will not be further condensed.

### 3.3. Common Factors of Both SP and LDs

Both SP and LDs are correlated to dream recall frequency [11,41]. Nightmare frequency also seems to be related to both SP and LDs [41,45]. However, Solomonova, Nielsen, and Stenstrom [49] did not find a connection between nightmares and LDs. Other dream-like phenomena connected to both LDs and SP were hypnagogic hallucinations, pleasant dreams [41], false awakenings, OBEs [11], and flying dreams [42]. However, LDs were positively correlated with flying dreams, and SP negatively.

Both were also connected to daydreaming, positive constructive daydreaming, dissociate experiences [43], delusional thinking, and reality testing [45].

LDs and SP are also connected to mental health, but seemingly in different ways: depression, anxiety, and stress were positively connected to SP, and negatively to LDs [43]. However, LDs were also connected to the use of antidepressants [41]. At the same time, SP was further connected to alcohol and nicotine. Alcohol has been found to be connected to stress and depression [50,51], and nicotine use is connected to stress and anxiety [52].

These trends were also found in other studies. LDs have often been connected to lower stress and better wellbeing overall [23,53], while SP is associated with worse mental health [54], higher self-reported measures of depression [55], and PTSD [56].

Another apparent connection was found between SP and LDs and paranormal phenomena, both being connected to paranormal experiences [45]; however, only SP was connected to paranormal belief, but LDs were connected to conspiracy belief in one study [43]. This is in accordance with previously described connections between both SP and LDs and the supernatural, such as the many culturally dependent figures made responsible for SP and the connection between SP and LDs and alien and UFO encounters.

Personality also played a role in LDs and SP, as both were connected to openness and extraversion [41]. Openness was further connected to pleasant SP [46]. 

Only SP was connected to sleep, especially sleep quality [43] and sleep time [11].

## 4. Discussion

As can be seen, there are many different connections between LDs and SP, such as the correlation and cooccurrences of these two phenomena, but also apparent interactions and common factors influencing both. In one of the studies mentioned above, SP was signaled using a specific eye movement. This is the technique that is normally used to detect LDs. Therefore, it seems to be functional in both and exemplifies, again, that experiencers of SP can voluntarily move their eyes. However, even though SP shares typical awareness with LDs and a feeling of being awake, it most often does not share the positive feelings that LDs are accompanied by. 

In the reviewed studies, only SP was connected to sleep quality. This is consistent with the current literature, as SP is known to be connected with reduced sleep quality (for an extensive review of the connection of SP and sleep quality, see [2]). For LDs, it was found that connections with reduced sleep quality disappear when nightmares are controlled for [57].

As stated above, OBEs are possible hallucinations in SP. Further, in these, SP is often filled with bliss and positive emotions. Herrero, Gallo, Gasca, Gleiser, and Forcato [4] argued that this might be due to the sense of agency, which is present in OBEs but mostly not present in other forms of SP. It has been discussed whether LDs and OBEs are, in fact, different phenomena, or at least if they partially overlap. As shown above, there is significant correlation between the two experiences. Further, as Herrero et al. [4] pointed out, the techniques used to induce OBEs are quite similar to those to induce LDs. Both of these phenomena are filled with bliss and positive feelings. In both, the experiencer takes an active role and can decide what to do next. Raduga et al. [11] argued that, at least in some cases, these two are the same phenomenon. 

It was argued that OBEs can be induced in patients who experience SP and help transform these into pleasant episodes [4]. Therefore, this could be another form of lucid dream and a way of overcoming SP. It has been shown that LDs can be effective in overcoming nightmares and changing them while they are happening [21,22]. This might also be applicable to SP, as it shares some common ground with nightmares [14,58]. Both are filled with fear, and nightmares can also include being paralyzed and physical harm [59]. Additionally, using LDs as a way to overcome the terrors of SP should mean that experiencers have less fright about dream figures and dream situations, which can lead to active confrontation, which may function as a specific form of mental hygiene [60].

Building on personal experience, Conesa [42] developed the method of Sleep Paralysis Signaling (SPS), which aims to use SP as a way to achieve LDs. It further aims at reducing fear and helplessness, which is normally experienced during SP. In a first step, the experiencer should focus their attention on a body part while breathing calmly. This alone might be enough to reduce fear and help the experiencer wake up or get back into regular dreams. In a next step, the experiencer should use techniques similar to those normally used to achieve LDs, for example, imagining the body spinning or falling and floating away. This might help transmit the SP into an LD. Conesa [42] speculated that this should result in an even greater awareness and control upon achieving lucidity. The technique can be combined with self-hypnosis techniques and meditation for better results. This can not only help the experiencer in the moment and let them enjoy an LD full of possibilities and bliss instead of a frightening SP, but it will also help them establish a better relationship with sleep in general, as they do not need to fear suffering from another SP but, instead, can happily await another change to “enjoy a remarkable otherworldly dreamscape” [42], p. 11. However, this technique still needs to be systematically tested to see if it is applicable to help various people overcome the negative aspects of SP. However, there are also other reports, in addition to Conesa [42], assuring us that LDs can, in fact, be used to handle SP. De la Brena and Schoenmann [61] also offered insights into the dreaming life of one of the authors who suffered from narcolepsy. They also used LDs as a way to handle terrifying dreams and SP.

SPS, however, is only one possible way to treat SP. There are currently both non-pharmacological and pharmacological ways to treat SP. Non-pharmacological treatments involve psychoeducation and sleep hygiene to improve sleep quality and reduce sleep fragmentation, as well as limiting substances that enhance the risk of SP, such as alcohol and nicotine. Psychopharmacological treatments involve normally used drugs, such as tricyclic antidepressants and selective serotonin reuptake inhibitors. These should repress REM sleep and have already been shown to reduce SP [1]. In order to assess SPS as a possible way to treat SP, it should also be compared to those other treatments.

For further research, it would also be interesting to find out why some people naturally experience OBEs from SP. As seen, at least part of these might be explainable through LDs and LD induction techniques, as these two are connected to pleasant SP, which is often connected to OBEs. However, it would be interesting to find out if some people can naturally achieve these and what characterizes them. Both LDs and SP include elements that are more associated with waking states than to dreaming states. Still, Mainieri et al. [48] argued, based on their findings, that the brain is not awake during SP, but it is in REM sleep. Although it is often suggested that SP occurs because muscle atonia is still active while the brain is awake, this might not be the case after all, and the brain is still in REM sleep during SP. This view is also shared by Hishikawa and Shimizu [62]. Similarly, LDs have recently been proposed to be happening in REM sleep and not during a “hybrid” state [31].

Another phenomenon, which is categorized as a dream but has increased awareness, is lucid nightmares [63,64]. These are nightmares in which the dreamer is aware that they are stuck in a dream, but they are not able to change the nightmares or escape them. Similarly, SP could also be a type of nightmare, resembling these lucid nightmares in the way that the experiencer is highly aware but trapped and mostly helpless during the episode. Whereas in SP, one has the feeling of being awake and mostly a very clear perception of what is going on around them, in lucid nightmares, they know they are dreaming and very clearly perceive their dream surroundings. The most important difference is that, in SP, the experiencer has the feeling of being awake and to be in the same place they fell asleep, while in lucid nightmares, the experiencer is aware of dreaming. In both cases, there is a lack of agency, and the experiencer feels helpless and “trapped”. We propose that those dreams, where the dreamer is aware of dreaming but has no control of the dream, or awakes upon this realization, should be called “pre-lucid” dreams, while those dreams where the dreamer has both the awareness of being in a dream and control over the dream should be called “lucid”. In this terminology, both SP and lucid nightmares can be seen as pre-lucid, in which a heightened awareness is present but the dreamer cannot change what is happening. We hope that both of these states, which are unpleasant and connected to fear, could be changed with a further understanding of LDs and learning how to control dreams. In lucid nightmares, this might turn out to be the final puzzle stone for the dreamer to not only realize they are dreaming but to also be able to change the dream plot of the nightmares into pleasant dreams or simply to wake up. Additionally, the fact alone that LDs are possible might be enough to give them relief during the nightmare. In SP, this might similarly be the case. In a first step, realizing what is going on, that one is not in fact paralyzed and that the frightening figures and sounds are not real, might be a relief for the experiencer. Further, it might give them a chance to relax and relieve the fear, which, as pointed out by Conesa [42], might be enough to either completely wake up or go into regular dreams, and it might give them a change to use SP to move into an LD.

## 5. Limitations

As has been seen, there are only a few studies that have examined SP and LDs. Therefore, the data to draw conclusions from are limited. The diversity of research topics and exact aims of the different studies make it difficult to compare and summarize them. Further, the studies vary in their methodologies used. However, these differences might also be a sign of the robustness of the connection between SP and LDs, as it shows up while using various methods.

Almost all the studies were conducted in Westernized countries, so generalization across different populations is difficult. However, this was not explicitly stated in most of the papers. Only two of the studies described where participants came from, with one where most were from Germany [41] and another, where most were from the USA, Canada, and the UK [42]. However, given the sampling strategy, it seems reasonable to assume that most are from similar countries as the affiliation of the authors, if not stated otherwise. Regarding cultural backgrounds, there is similarity in all the countries examined. One study specifically compared narcolepsy patients with healthy subjects, but other sleep disorders were not examined. Additionally, most of the participants in the different studies were adults, so the findings might not generalize to children and adolescents.

Another drawback is that most of the studies used self-selected samples and samples with participants who were interested in the topics. As seen above, LD-inducing techniques can also invoke SP, so the correlations seen in the studies might be inflated, as the participants are probably interested in LDs and might have tried to induce them and induce SP.

## 6. Conclusions and Possible Lines of Future Work

There is a connection between LDs and SP; however, its exact details still need to be explored. This does not only include the correlation between these two phenomena but also other factors that interact with those. There is still very limited research, so possible conclusions are limited. This emphasizes the need to replicate the findings of the found studies and generalize them in different samples. Further, a standardized way to research these phenomena is needed to better compare different studies and better generalize the results. Although various questionnaires have already been introduced, to ensure there are advances in the field, it is necessary to have comparability between various studies. This could be achieved by either developing a universal tool to measure LDs or SP but also in examining the different existing measures and questionnaires in order to make their results comparable.

Further, possible lines of future work include systematically assessing if LDs can be used to overcome SP and in which cases this would be applicable. Additionally, it would be interesting to examine who naturally experiences OBEs and LDs during SP and what mechanisms support the natural transition between those states.

## Figures and Tables

**Table 1:** Summaries of the different studies, with the citation, the design of the study, the sample-size, what was measured, and the main results.

Study	Design	Sample-Size	Measures	Main Results
Biehl, 2022 [41]	Online Survey	436 (45.7% female)	Frequency of LD, SP, and other dream phenomena. Intake of food and substances. Personality	LD and SP correlated with r = 0.276, *p* < 0.001, and were both connected to other dream phenomena.
Conesa, 2002 [42]	Case Study	1	Noted dreams and dream related phenomena	LD and SP correlated with r = 0.31. Both were further connected to flying dreams
	Online Survey	92 (not reported)	Not reported	16.3% report experiencing both LD and SP
Denis, & Poerio, 2017 [43]	Online Survey	1928 (53% female)	Frequency and intensity of SP, LD, and hallucination types of SP. Various other measures	LD correlated with SP r = 0.15, *p* < 0.001, intruder hallucinations, and V-M hallucinations. Both were also connected to various other measures
Dodet, Chavez, Leu-Semenescu, Golmard, & Arnulf, 2015 [44]	Case-Control Study using Interviews	53 + 53 = 106 (41.5% and 43.3% female)	In-Person interviews about nighttime sleep characteristics, such as SP, LD and others	Participants with narcolepsy had both LD and SP more often than those without narcolepsy (58.5% vs. 17% and 58.5% vs. 15.1%)
Drinkwater, Denovan, & Dagnall, 2020 [45]	Online Survey	455 (76% female)	Frequency of LD. Overall recall of SP, Nightmares, Nightmare distress, reality testing deficits, paranormal experience and belief	LD and SP correlated with r = 0.23, *p* < 0.01. Both were connected to Nightmare frequency, paranormal experience, delusional thinking, and reality testing
Kliková, Sharpless, & Bušková, 2021 [46]	Online Survey	172 (68% female)	Frequency of SP. Occurrence of LD, Ability to induce LD, Questions on pleasant SP, for example occurrence and frequency. Trauma, life satisfaction, Personality	Positive association between pleasant SP and LD: X2 (1, N = 172) = 8.414, *p* = 0.004, φ = 0.22 and pleasant SP and the ability to induce LD: X2 (1, N = 134) = 9.327, *p* = 0.002, φ = 0.26
LaBerge, 2004 [47]	Randomized trial	10 (30% female)	Dream content, other self-reported measures of nightly sleep and dreams	Donepezil was associated with both LD and SP
Mainieri et al., 2021 [48]	Observational EEG study	5 (80% female)	EEG, EOG, EMG	All episodes of SP happened during REM sleep. Increased alpha rhythms in SP compared to LD but less enhanced muscle tone.
Raduga, Kuyava, & Sevcenko, 2020 [11]	Live Survey	974 (54% female)	Frequency of LD, SP, dream recall frequency, false awakenings, OBE. Sleep duration, overall awareness of practices such as LD	Positive association between LD and SP frequency, X2 (25, N = 974) = 126.767, *p* < 0.001, φ = 0.36 Both connected to dream recall frequency, false awakenings, OBE.
Solomonova, Nielsen, & Stenstrom, 2009 [49]	Online Survey	245 (58% female)	Frequency of LD, SP, nightmares. SP-, and Nightmare distress	LD correlated with SP r = 0.24, *p* < 0.001, and SP r = 0.21, *p* = 0.001

LD: lucid dreaming, SP: sleep paralysis.

## Data Availability

Not applicable.
